# INVOLVING PEOPLE WITH A FRONTOTEMPORAL DEMENTIA DIAGNOSIS IN DEMENTIA RESEARCH

**DOI:** 10.1093/geroni/igac059.141

**Published:** 2022-12-20

**Authors:** Martina Roes, Marie-Marleen Heppner, Sonja Teupen, Jan Oyebode, Claus Heislbetz, Vanaditz Goetz, Lara Pivodic, Lauren Massimo

**Affiliations:** German Center for Neurodegenerative Diseases (DZNE), Witten, Nordrhein-Westfalen, Germany; German Center for Neurodegenerative Diseases (DZNE), Witten, Nordrhein-Westfalen, Germany; German Center For Neurodegenerative Diseases (DZNE), Witten, Nordrhein-Westfalen, Germany; Bradford University, Bradford, England, United Kingdom; Hans-Weinberger-Akademie der AWO e.V., Muenchen, Bayern, Germany; Hans-Weinberger Akademie der AWO e.V., Muenchen, Bayern, Germany; Vrije universiteit Brussel, Ixelles, Brussels Hoofdstedelijk Gewest, Belgium; University of Pennsylvania, Philadelphia, Pennsylvania, United States

## Abstract

Frontotemporal Lobar Degeneration (FTLD) is a form of dementia that is characterized by prominent and gradual changes in behavior and language, whereas memory is relatively preserved (therefore different from Alzheimer dementia). In our FTD project we analyze care services for people with FTLD in Germany and we are working together with persons with FTLD and their partners as patient advisors. Our presentation will reflect on how we communicate (e.g. easy Language), how we communicate with the dyads (e.g. balance within the dyads), how we prepare for and engage during meetings as well as how this influences our research project. In summary, building trust and staying connected between meetings, being transparent about expectations and applying a narrative approach seems to be a way to successfully involve people with FTLD and their partners as advisors in dementia research.

